# MicroRNA-375: potential cancer suppressor and therapeutic drug

**DOI:** 10.1042/BSR20211494

**Published:** 2021-09-22

**Authors:** Jiahui Wei, Yiran Lu, Ruiqing Wang, Xiangzhu Xu, Qing Liu, Song He, Huihao Pan, Xinmiao Liu, Bao Yuan, Yu Ding, Jiabao Zhang

**Affiliations:** 1 Department of Laboratory Animals, College of Animal Sciences, Jilin University, Changchun, Jilin 130062, P.R. China; 2 The Eye Center in the Second Hospital of Jilin University, Ziqiang Street 218#, Nanguan District, Changchun City 130041, Jilin Province, China; 3 Department of Laboratory Animals, College of Animal Medicine, Jilin University, Changchun, Jilin 130062, P.R. China; 4 The Second Clinical School of Medicine, Guangdong Provincial Hospital of Chinese Medicine, Guangdong Provincial Hospital of Chinese Medicine-Zhuhai Hospital, Guangzhou University of Chinese Medicine, Guangzhou 510120, Guangdong, China

**Keywords:** Dysregulation, EMT, miR-375, Tumor-suppressive

## Abstract

MiR-375 is a conserved noncoding RNA that is known to be involved in tumor cell proliferation, migration, and drug resistance. Previous studies have shown that miR-375 affects the epithelial–mesenchymal transition (EMT) of human tumor cells via some key transcription factors, such as Yes-associated protein 1 (YAP1), Specificity protein 1 (SP1) and signaling pathways (Wnt signaling pathway, nuclear factor κB (NF-κB) pathway and transforming growth factor β (TGF-β) signaling pathway) and is vital for the development of cancer. Additionally, recent studies have identified microRNA (miRNA) delivery system carriers for improved *in vivo* transportation of miR-375 to specific sites. Here, we discussed the role of miR-375 in different types of cancers, as well as molecular mechanisms, and analyzed the potential of miR-375 as a molecular biomarker and therapeutic target to improve the efficiency of clinical diagnosis of cancer.

## Background

MicroRNAs (miRNAs; 20–24 nucleotides) are a type of small endogenous RNA that act as post-transcriptional regulators of gene expression [1]. Interestingly, each miRNA can have multiple target genes, and each gene can be regulated by several miRNAs [2]. Additionally, they have been shown to be involved in the pathogenesis of many diseases [3]. Cancer has become one of the leading causes of deaths worldwide [4]. Previous studies have shown that miRNA dysregulation is the main cause behind the onset of various cancers [5]. Since miRNAs might act as a tumor suppressor or oncogenes, their function in carcinogenesis is frequently investigated in clinical studies to find therapeutic targets [6]. MiR-375 is known to be dysregulated in various cancers, where it usually acts as a tumor suppressor factor [7]. Jia-yuan et al. showed that overexpression of miR-375 inhibited the development of nasopharyngeal carcinoma (NPC) [8]. Transcriptional enhanced associate domain (TEAD) is the main binding partner of Yes-associated protein 1 (YAP1) and acts as a transcriptional activator in various cancers [9]. Due to promoter methylation and histone deacetylation, the expression of miR-375 in human gastric cancer has been shown to decrease. The clinical and correlation analysis of primary gastric cancer showed that miR-375 targeted the three components of the Hippo pathway, YAP1, TEAD4, and connective tissue growth factor (CTGF), to exert an anti-cancer effect [10].

**Figure 1:**
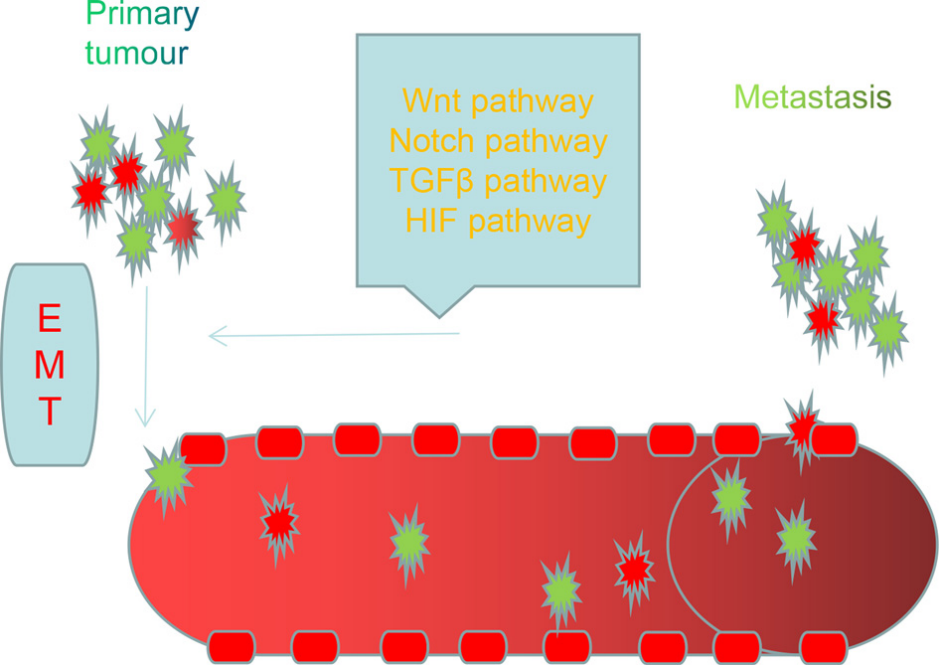
The activation of EMT EMT-related signaling pathways are essential for the occurrence of EMT. EMT allows tumor cells to invade and metastasize and may also allow tumor cells to escape apoptosis.

Epithelial–mesenchymal transition (EMT) refers to the process of transformation of epithelial cells into cells with a mesenchymal phenotype [11]. In the process of EMT, epithelial cells lose cell polarity, lose connection with the basement membrane, as well as gain increased migratory and invasive abilities, anti-apoptotic and extracellular matrix-degrading capacity [12] (Figure 1). Epithelial tumor cells lose the ability to adhere and undergo mesenchymal cell migration to promote metastasis and drug resistance [13]. Wnt, nuclear factor κ B (NF-κB) and transforming growth factor β (TGF-β) signaling pathways have been shown to be closely related to tumor EMT [14–16]. Studies have shown that miR-375 participates in EMT via key proteins or transcription factors involved in these pathways, thereby affecting tumorigenesis [17]. For example, miR-375 has been shown to directly target E-cadherin to enhance EMT of human cervical cancer cells [18]. Additionally, miR-375 has been shown to inhibit the migration and invasion of human colorectal cancer (CRC) cells by altering the expression of EMT-related proteins [19].

**Figure 2:**
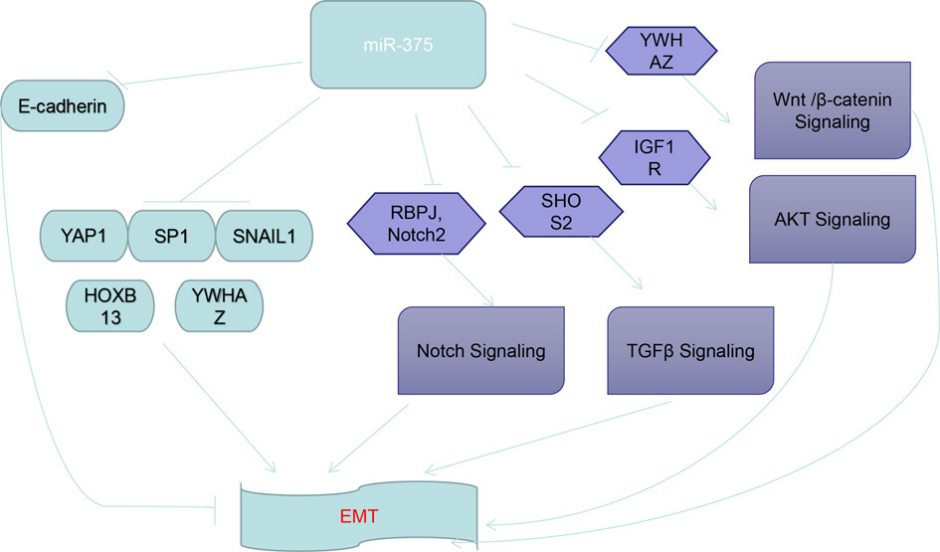
Schematic diagram of the mechanism of miR-375 in EMT MiR-375 attenuates EMT by inhibiting vital target genes (such as *YAP1, SP1, SNAIL1, HOXB13, YWHAX*). EMT can also be enhanced by inhibiting E-cadherin. Additionally, some signaling pathways related to EMT also act as intermediaries between miR-375 and EMT. For example, miR-375 inhibits the expression of RBPJ and Notch2 to inhibit the NOTCH pathway, while miR-375 also inhibits the WNT pathway by reducing the expression of YWHAZ. Also, miR-375 reduces the activity of the TGF-β pathway by inhibiting the expression of SHOS2. Finally, miR-375 inhibits the AKT signaling pathway by inhibiting the level of IGF1R, regulating EMT.

Here, we discuss the role of miR-375 in the development as well as in the treatment of various cancers (Figure 2).

## The role of miR-375 in CRC

Studies have shown that the expression of miR-375 in human CRC tissues and cell lines are significantly reduced compared with paracancerous tissues [20]. Specificity protein 1 (SP1) is a transcription factor involved in the apoptosis, proliferation, and differentiation of cancer cells [21]. The overexpression of miR-375 has been shown to inhibit the proliferation of human CRC cells by directly binding to the target gene, *SP1* [22]. On the other hand, miR-375 has also been shown to exert a synergistic anti-cancer effect with other drugs, along with an increase in the drug sensitivity of cancer cells [23]. Xueni et al. found that overexpression of miR-375 increased the *in vitro* anti-CRC effects of 5-fluorouracil (5-FU) and cetuximab [24]. Therefore, miR-375 might act as the molecular biomarker for chemoresistance in patients with CRC. miR-375 is also known to be involved in epigenetic anti-cancer effects [25]. METTL14 has been shown to reduce the level of m6A methylation through the miR-375/SP1 pathway to inhibit the development of human CRC cells [26]. The metastasis of CRC is vital for treatment because the metastasis of malignant tumors increases the damage to the body, leading to treatment failure, and the EMT of CRC is vital for the metastasis process [27]. As a tumor suppressor gene, miR-375 has been shown to inhibit the metastatic ability of CRC cells by altering the expression of EMT-related genes [19]. Campayo et al. analyzed the RNA in the microscopic tissues of 96 patients with rectal cancer who were treated with preoperative chemoradiotherapy (CRT) and found that the combination of miR-99b and miR-375 was beneficial for patients with rectal cancer [28]. Additionally, in a DLD-1 tumor model, Coxsackie virus B3 (CVB3) with miR-375 inserted could effectively fight CRC and reduce the toxicity to the pancreas and heart [28]. Thus, these results proved that miR-375 could effectively inhibit the development of CRC.

## The role of miR-375 in prostate cancer

Prostate cancer (PC) is caused by the presence of malignant tumor lesions in the epithelium of the prostate [29]. It is known to primarily occur in 70–80-year-old males [30]. Simin et al. analyzed the TCGA database and confirmed that compared with human adjacent non-cancerous tissues, the expression of miR-375 in PC tissues was significantly enhanced [31]. Additionally, bioinformatics analysis showed that the target genes of miR-375 were mainly involved in gene transcription and ubiquitin-mediated proteolysis to inhibit PC cell migration and EMT [31]. Xiaoyi et al. analyzed the blood samples of 100 patients with PC and found that there was a significant association between their survival rate and the expression of miR-375, which might act as a prognostic biomarker for PC [32]. Additionally, the *in vivo* xenograft mouse model showed that the miR-375-transfected group showed a reduced effect of docetaxel treatment, which was probably due to the role of miR-375 and target genes in the development of drug resistance [33]. It is also possible to distinguish between patients with PC and benign prostate hyperplasia (BPH) by analyzing the levels of miR-375 in the blood to achieve symptomatic treatment [34]. On the other hand, DNA methylation is known to play a key role in inhibiting gene expression, and the expression of Androgen receptors (AR) and total DNA methyltransferases (DNMTs) can regulate the expression of miR-375 by altering the methylation of the miR-375 promoter [35].

## The role of miR-375 in breast cancer

Breast cancer (BC) cell EMT plays an important role in the recurrence and metastasis of BC [36]. Although there are many types of BC cells (Luminal A, Luminal B, HER2 enrichment, Basal-like, and Claudin-low), there is an elevated expression of miR-375 in ER-positive cells compared with ER-negative cells, mainly caused by cell differentiation [37]. Based on the results of experiments in zebrafish and cell lines, in MDA-MB-231 and Hs578T cells, miR-375 inhibits EMT by inhibiting the expression of stature homeobox 2 (SHOX2), which is known to activate the TGF-β signaling pathway [38]. Studies have shown that signal transducer and activator of transcription 3 (STAT3) is overactivated in various types of BCs [39]. Overexpression of Janus kinase 2 (JAK2) can enhance the stemness of BC cells and rescue the suppression of BC cell stemness induced by miR-375, miR-375 uses JAK2/STAT3 pathway to enhance the sensitivity of adriamycin and reduce its resistance [40]. The up-regulated expression of hsa-miR-375 accelerates cell proliferation by regulating the PI3K/Akt/mTOR pathway, and the overexpression of Ras-related dexamethasone-induced 1 (RASD1) reduces the activity of the Akt/mTOR pathway [41]. Zellinger et al. analyzed 53 BC patients and found that patients with low miR-375 expression were more likely to have a local recurrence, which was probably related to RASD1 signaling [42]. Additionally, miR-375 was also found to be involved in the growth of fulvestrant-resistant breast cancer (FRBC) cells and reducing autophagy [43].

## The role of miR-375 in lung cancer

Histologically, lung cancer (LC) is divided into non-small cell lung cancer (NSCLC) and small cell lung cancer (SCLC) [44]. The NSCLC subtypes are further divided into squamous cell carcinoma (SCC) and adenocarcinoma (AC) [45]. Due to the stable expression of miRNA in plasma, a study tested 38 plasma samples from patients with SCLC and found that the expression of miR-375 was significantly increased, which might have been caused by the activation of the transcription factor achaete-scute-1 (ASCL1) [46]. Interestingly, the expression of miR-375 was found to be negatively related to the patient survival rate but was not related to the patient’s age [47]. Yu et al. found that compared with healthy subjects, plasma miR-375 levels in NSCLC patients were significantly lower, and the patients with high miR-375 expression had a higher survival rate [48]. Additionally, miR-375 has been shown to enhance the sensitivity of NSCLC to radiation, thereby inhibiting tumor growth [49]. On the other hand, Yoda et al. found that miR-375, which is known to be highly expressed in clinical samples of NSCLC, had stronger migration and invasion capabilities, which shortened the survival period of patients; this was potentially due to the difference in the internal tumor microenvironment [50]. Also, the results of tissue biopsy showed that miR-375 could also be used as a marker to identify SCC and AC, with approximately 96% accuracy [51]. These results could be used as prognostic indicators of miR-375.

## The role of miR-375 in osteosarcoma

Osteosarcoma (OS) occurs mostly in children and adolescents [52]. MiR-375 has been shown to inhibit the proliferation of OS cells. The Kaplan–Meier survival curve of the TCGA sarcoma dataset showed that patients with elevated expression of miR-375 had a higher 10-year survival rate [53]. Drug resistance has been shown to be one of the biggest obstacles to the treatment of OS [54]. Gao et al. found that the expression of miR-375 was down-regulated in diamine dichloro platinum (DDP)-resistant OS tissues and U2OS and MG63 cells. In a mouse xenograft model, miR-375 was found to reduce the growth of tumor cells, which was probably related to the mediation of ATG2B or Mcl-1 [55]. The results of Cell Counting Kit-8 (CCK8) and flow cytometry assays found that circ-0060428 inhibited the growth of U2OS and HOS cells and promoted their apoptosis by sponging miR-375, which could be a novel idea for the treatment of OS [56]. However, on the other hand, Hu et al. found that OS was probably hormone-dependent cancer [57]. Studies have shown that phytoestrogen formononetin has the potential to reduce the expression of miR-375 in U2OS cells to inhibit tumor growth, anti-hormone therapy could also be an effective mode of therapy for OS [57].

## The role of miR-375 in hematologic neoplasm

The expression of miR-375 has been found to be down-regulated in some hematomas [58]. Compared with normal, the expression of miR-375 in patients with multiple myeloma (MM) was significantly reduced, and the introduction of miR-375 down-regulated PDPK1 in human myeloma cell lines (HMCLs), which probably affected the development of MM through phosphoinositide-dependent protein kinase 1 (PDPK1)/ribosomal protein S6 kinase A3 (RPS6KA3). [25]. Acute myeloid leukemia (AML) is a malignant disease of the myeloid hematopoietic stem/progenitor cells with a higher rate of incidence in infants and young children [59]. Compared with normal hematopoietic stem cells, the expression of miR-375 is generally reduced in the leukemia cell line HL-60, and its low levels are related to the poor prognosis of patients [60]. Additionally, the overexpression of miR-375 was found to reduce the expression of HOXB3, and knockdown of HOXB3 reduces the expression of cell division cycle associated 3 (CDCA3), thereby inhibiting the proliferation of HL-60 cells [60]. These indicate that increasing the levels of miR-375 might be a potential strategy to treat leukemia [61].

With the development of genome sequencing technology, more and more sequencing results have shown that miR-375 plays a role in inhibiting tumor growth, migration, invasion, and EMT process [62]. In addition to the above-mentioned cancer types, we have also summarized the imbalance of miR-375 in other cancers (Table 1).

**Table 1:** The expression of miR-375 in different human cancers

Tumor type	miR-375	Number of tumor samples	Compared with normal tissues	Reference
ESCC	miR-375	50	Down	[116]
ESCC	miR-375	40	Down	[117]
CRC	miR-375	40	Down	[24]
CRC	miR-375	112	Down	[26]
HCC	miR-375	30	Down	[58]
HCC	miR-375	53	Down	[83]
HNSCC	miR-375	520	Down	[118]
HNSCC	miR-375	123	Down	[119]
LSCC	miR-375	50	Down	[120]
LSCC	miR-375	40	Down	[92]
MTC	miR-375	130	Down	[121]
MTC	miR-375	9	Down	[122]
OC	miR-375	50	Up	[123]
OSCC	miR-375	40	Down	[124]
OSCC	miR-375	40	Down	[125]
OS	miR-375	42	Down	[126]
OS	miR-375	30	Down	[68]
ADCC	miR-375	15	Down	[127]
BC	miR-375	10	Down	[128]
BC	miR-375	50	Down	[129]
SCLC	miR-375	63	Up	[47]
PC	miR-375	495	Up	[35]
PC	miR-375	146	Up	[34]
GC	miR-375	49	Down	[71]
NPC	miR-375	38	Down	[8]

Abbreviations: ADCC, adenoid cystic carcinoma; COAD, colon adenocarcinoma; ESCC, esophageal squamous cell carcinoma; GC, star carcinoma; HCC, hepatocellular carcinoma; HNSCC, head and neck squamous cell carcinoma; LAC, lung adenocarcinoma; LSCC, laryngeal squamous cell carcinoma; MTC, medullary thyroid cancer; NB, neuroblastoma; OC, ovarian cancer; OSCC, oral squamous cell carcinoma; PTC, papillary thyroid carcinoma.

## The upstream regulatory mechanism of miR-375

There is a lack of research data on the upstream regulators of miRNA. In addition to some proteins, there are some noncoding miRNAs, such as lncRNA and circRNA, which regulate miRNA [63]. Chang ye Li et al. established a pulmonary fibrosis model in bleomycin (BLM)-induced C57BL/6 mice, compared with the control group, the lung tissue of the model group was sequenced, and a total of 85 mRNAs, 11 miRNAs, and 10 lncRNAs were co-expressed and were found to interact with each other [64]. Thus, the regulatory relationship of these RNAs provided a new method for the treatment of pulmonary fibrosis [65]. These factors also act as the upstream regulators of miR-375. Thus, understanding the effects of these factors and the associated mechanism of action might facilitate the use of miR-375 for treatment. Both lncRNAs and circRNAs are noncoding RNAs that are >200 nt and are found in the cytoplasm or the nucleus [66]. They are known to regulate various important life activities [67]. Additionally, they also form miRNA sponges, which prevent miRNAs from binding to target genes in various nude mouse models of cancer cells and tumors [64]. Wang et al. found that compared with normal tissues of OS, there was an abnormally high expression of lncRNA tumor suppressor candidate 7 (TUSC7) in OS tissues, and lncRNA TUSC7 could combine with miR-375 to inhibit the proliferation and migration of OS cells [68]. LncRNA-small nucleolar RNA host gene 17 (SNHG17) was found to be elevated in colon adenocarcinoma (COAD) tissues and cells [69]. The miR-375 participated in the COAD cell behavior regulated by SNHG17, and the overexpression of chromobox 3 (CBX3) promoted the malignant behavior of COAD cells. The results of the dual-luciferase assay showed that miR-375 was the target of (SNHG17) and CBX3 was the target of miR-375 [70]. The results of the mouse xenogeneic inhibition model showed that the overexpression of miR-375 could inhibit the proliferation of lncRNA-SNHG17 in COAD cells, thus, SNHG17/miR-375/CBX3 regulated the development of COAD [70]. Additionally, differentially expressed circRNAs were screened by microarray chip on human GC tissue and normal tissue, and the results showed that hsa_circ_0008365 (circ-SERPINE2) was highly expressed in GC tissues [71]. Tyrosine 3-monooxygenase/tryptophan 5-monooxygenase activation protein ζ (YWHAZ) was used as a clinical prognostic indicator for some tumors [72]. In human gastric cancer cells (AZ521, MGC-803), circ-SERPINE2 could competitively bind to miR-375 to affect the transcription of YWHAZ, thereby promoting the development of gastric cancer [71]. Thus, these results indicated that lncRNAs and circRNAs affected the role of miR-375 in tumors via the ceRNA mechanism. In addition to the above upstream regulators, Table 2 summarizes other upstream genes of miR-375.

**Table 2:** The interaction between upstream regulators and miR-375

Tumor type	Upstream regulator	Number tumor samples	Compared with normal tissues	Reference
Bladder cancer	circKIF4A	50	Up	[129]
OS	CircFAT1	12	Up	[53]
OS	Circ 0060428	Four cells	Up	[56]
ESCC	circLPAR3	50	Up	[116]
GC	lncRNA MLK7-AS1	5	Up	[130]
GC	lncRNA TINCR	56	Up	[131]
GC	hsa_circ_0008035	30	Up	[132]
HCC	CircZFR	40	Up	[133]
SCLC	Circ_0086720	52	Up	[49]
HCC	Hsa_circ_101280	21	Up	[134]
CRC	Circ-PRKDC	30	Up	[135]
Cervical cancer	circEPSTI1	Two cells	Up	[136]
OS	lncRNA TUSC7	30	Up	[68]
CRC	lncRNA-SNHG17	45	Up	[70]
Neuroblastoma	lncRNA XIST	36	Up	[137]
HCC	LncRNA CCAT1	Two cells	Up	[138]
Gliomas	lncRNA RP11‐626G11.3	42	Up	[139]
PTC	LINC02471	50	Up	[88]
liver cancer	lncRNA MALAT1	20	Up	[140]
Glioma	lncRNA KCNQ1OT1	43	Up	[141]
Glioma	lncRNA LUCAT1	38	Up	[142]
LAC	lncRNA ROR1-AS1	50	Up	[89]
OSCC	LncRNA SNHG17	40	Up	[125]

## Mechanism of miR-375 in cancer metastasis

One of the biggest challenges in the treatment of cancer is to solve the problem of cancer cell metastasis and invasion [73]. EMT refers to the transition from epithelial cell type to mesenchymal cell type [74]. EMT is known to be related to tumor stemness, metastasis, and resistance to treatment, and is an essential factor for tumor invasion and distant spread and thus, plays an important role in the development of cancer (Figure 1) [75]. The regulation of EMT is a complex network, which includes the TGF-β family, Wnt signaling, Notch, Smad, HGF, FGF, and HIF, and other signaling pathways [76]. Recent studies have shown that miR-375 inhibitor treatment can promote EMT of human GC cells, which, in turn, affects carcinogenesis [77].

E-cadherin is known to play a vital role in the selective aggregation of cells during growth and development [78]. The E-cadherin-mediated cell adhesion and loss of cell connections can cause cells to separate from the primary tumor and migrate to distant sites [79]. Studies have shown that miR-375 can bind to E-cadherin 3′-UTR to enhance the EMT of human cervical cancer cells (SiHa, CaSki) [18]. The reversal of miR-375 or E-cadherin expression has the potential to overcome paclitaxel-induced drug resistance in human cervical cancer [18]. Additionally, miR-375 also has the potential to inhibit the invasion and migration of various PC cell lines by interacting with the transcription factor YAP1, but the EMT transcription factor zine finger E-box-binding protein (ZEB1) can also inhibit the transcription of miR-375 [32]. Also, the Sp1 transcription factor (SP1) is a ubiquitous nuclear transcription factor that is important for tumorigenesis [80]. SP1 is known to be a downstream target of miR-375, so, miR-375 targets SP1 to inhibit EMT-related genes, such as matrix metalloproteinase 2 (MMP2), vimentin, snail, β-catenin, N-cadherin, thereby inhibiting the migration and invasion of human CRC cells (DLD1, HCT8) [19]. HOXB13 is a highly conserved transcription factor; the abnormal expression of HOXB13 is related to carcinogenesis [81]. The overexpression of HOXB13 has been shown to lead to the formation of the CSC phenotype and the enhancement of EMT of MCF-7 cells [37]. The results of the dual-luciferase assay verified that miR-375 could inhibit the expression of HOXB13 and inhibit the migration and invasion of human BC cells and tamoxifen resistance [37]. Recent studies have shown that achaete-scute homolog-1 (ASH1) is the upstream regulator of miR-375 in human LC cells [82]. The reduced expression of YWHAZ, a downstream gene of miR-375, has been shown to be a significant relationship with the healing rate of patients and the decrease in EMT, ASH1-miR-375-YWHAZ acts as a signaling axis that affects human LC cells [83]. De et al. showed that human ovarian cancer cells with low E-cadherin expression had a stronger migration and invasion ability [84]. The EMT factor SNAIL1 has been shown to inhibit the transcription of E-cadherin [85]. The results of *in vitro* and *in vivo* experiments have shown that miR-375 attenuates the EMT of SKOV3 cells by targeting the expression of SNAIL1 since miR-375 is involved in the tumor microenvironment [86]. It has been reported that human papillary thyroid carcinoma (PTC) tissues and cell lines exhibit a reduced expression of miR-375 [87]. Inhibiting the expression of miR-375 has been shown to promote the proliferation and invasion of human PTC cell lines and EMT [88]. After the overexpression of miR-375, the results were found to be contradictory, but the specific mechanism needs further elucidation [88]. In addition, long noncoding RNA ROR1-AS1 (ROR1-AS1) has been shown to attenuate EMT in human lung adenocarcinoma (LAC) cells through the miR-375 sponge, which might be a novel idea for the treatment of LAC [89].

In addition, miR-375 regulates the EMT process not only through some genes but also through some signaling pathways [90]. For example, miR-375 has been shown to inhibit the migration, invasion, and EMT of human GC cells by targeting YWHAZ (14-3-3ζ) and inhibits the activation of the Wnt/β-catenin signaling pathway [77]. Previous studies have shown that the protein kinase B (AKT) signaling pathway acts as the upstream pathway of the EMT process in several types of human tumor cells [91]. In human LSCC cells, miR-375 has been shown to regulate the protein expression of E-cadherin, vimentin, and Snail2 through the AKT signaling pathway to inhibit cell migration and invasion, while IGF1R acts as a negative regulator of the AKT signaling pathway [92]. Studies have also shown that miR-375 can reduce the protein expression of Short SHOX2 and inhibit the proliferation of human BC cells, many BC cell lines have shown that SHOX2 acts as an inducer of EMT and also acts as a TGF-β receptor I (TβR-1) transcription factor that affects EMT through the TGF-β signaling pathway [38]. Additionally, miR-375 is known to be significantly decreased in highly aggressive human MCC cells, and silencing miR-375 has been shown to inhibit the expression of the Notch pathway-related proteins (Notch2 and RBPJ) and promote EMT [93]. However, the results of some studies have shown that almost complete knockdown of miR-375 in human MCC cell lines has no effect on cell development [94]. Also, it did not cause obvious effects on oncogenic signaling pathways (Hippo and EMT), which means that miR-375 is unlikely to act as a tumor suppressor gene in MCC cells [95]. This was probably due to the fact that there were other long noncoding RNAs of spongy miR-375 in cancer cells (Figure 2).

## MiR-375 as a potential diagnostic or prognostic biomarker

Since miR-375 is abnormally expressed in various cancers, it is possible to predict carcinogenesis by detecting the expression of miRNA in the cell. According to previous studies on the role of miR-375 in cancer, an attempt was made to use miR-375 as a tool for diagnostic or prognostic purposes. For example, the reduced expression of miR-375 in human liver cancer was found to be related to lymph node metastasis and TNM staging, and could act as a reference biomarker for liver cancer diagnosis [96]. In addition, miR-375 could be used as a biomarker to predict the prognostic survival time of patients with esophageal cancer (EC) in areas with a high incidence in China, and was found to be directly proportional to the survival rate of patients [97]. The level of miR-375 was also related to the stage of cancer. The study found that in human gliomas, miR-375 expression decreased, and the pathological grade worsened; thus, it could be used as a poor prognostic marker of human gliomas [98]. In the other cancers, miR-375 also acted as a potential biomarker for chemoresistance response. For example, the expression of miR-375 was significantly elevated in human small cell lung cancer (SCLC) chemoresistance cells, and it decreased after effective chemotherapy. It could be used to monitor changes in resistance of SCLC patients during chemotherapy [99]. The study found that increased expression of miR-375 in human metastatic castrate-resistant prostate cancer (mCRPC) cells led to a decrease in the chemotherapeutic effect of Docetaxel, highlighting the role of miR-375 as a potential biomarker of chemotherapy resistance in the mCRPC stage [35].

The impairment of blood glucose metabolism is known to lead to an impaired cell respiration and increased anaerobic glycolysis, thereby causing normal cells to become cancerous. In addition, miR-375 is an important miRNA in pancreatic islet development. It can participate in glucose homeostasis by affecting the secretion of insulin. For example, the overexpression of miR-375 in MIN6 cells could inhibit glucose-induced insulin secretion. Thus, miR-375 could be a potential biomarker for the treatment of diabetes [100]. In cancer cells, glucose is preferentially metabolized through aerobic glycolysis, increasing the production of glycolysis and lactic acid. Studies have shown that miR-375 often acts as a tumor suppressor in human HNSCC cancer cells possibly due to is the fact that the overexpression of miR-375 promotes insulin secretion, improving blood glucose metabolism in HNSCC patients; thus, it could be used as a prognostic survival marker for HNSCC patients [101].

Although there are many studies on the use of miR-375 as a potential diagnostic or prognostic biomarker, clinical sample data still expediate the process of using miR-375 as a clinical biomarker.

## MiR-375 is expected to be used in clinical treatment

MiRNA is a type of noncoding single-stranded RNA encoded by endogenous genes. One miRNA can regulate multiple target genes and pathways, with minimal side effects and better clinical treatment effects. miR-375 acts as a tumor suppressor in most cancers. The *in vivo* or *in vitro* regulation of miR-375 expression through miRNA mimics or inhibitors is being explored as a novel type of therapy [102]. For example, the transfection of human NPC cells with miR-375 mimic resulted in a significant reduction in their migration and invasion ability [103]. The miR-375 overexpressing human BC cells constructed by lentivirus infection were found to not only reduce CSCs but also reduce the resistance of human BC cells to adriamycin [40]. Another study found that stable transfection of miR-375 precursors into human CRC cells could reverse the drug resistance of CRC cells in nude mice, inhibiting tumor formation [104]. These observations provide a theoretical basis for the use of miR-375 analogs in the clinical treatment of cancer. However, further studies are needed to determine methods to efficiently, safely, and sustainably deliver miR-375 to target cells. Thus, we designed various effective delivery systems to improve the efficiency of targeting liposomes, exosomes, polymers, dendrimers, mesoporous silica nanoparticles, and metal-based nanoparticles [105]. Due to their greater intratumoral accumulation, enhanced endosomal escape ability, greater therapeutic effect, and reduced systemic toxicity, these nanoparticle-mediated miRNA delivery has become a universal and valuable carrier [105]. The polymer carrier offers slow-release, low toxicity, and difficult to degrade [106]. After entering the circulatory system of the body, it has been shown to prepare the drug to be delivered to the target [107]. Xue et al. found that overexpression of P-glycoprotein (P-gp) caused drug resistance in human liver cancer, and miR-375 inhibited the expression of P-gp [108]. Therefore, using lipid-coated hollow mesoporous silica nanoparticles (LH) containing doxorubicin hydrochloride (DOX) and miR-375 (LHD/miR-375) were introduced into liver cancer cells and tissues, *in vivo* and *in vitro* studies found that LHD/miR-375 effectively inhibited the growth of tumors without producing significant toxicity [108]. In addition, miR-375 and DOX co-loaded into lipid-encapsulated calcium carbonate nanoparticles (LCC-DOX/miR-375 NPs) showed joint anti-tumor ability in xenograft mice and reduced the resistance of human LCC [109]. However, nanoparticle carriers were also found to have a certain restrictive effect; they could also lead to immunogenicity and toxicity. Therefore, there is an urgent need to continuously optimize the delivery vector of miRNA for better clinical application in the future [110].

## Conclusion

The primary cause that limits effective cancer treatment is the mutation of cancer cells, genome instability, and mutations, which can change the fate of cancer cells and accelerate the migration and invasion of cancer cells, which can indefinitely proliferate and destroy the function of normal organs [111,112]. MiR-375 is known to act as a tumor suppressor in most tumor cells (Table 1) and plays an important role in tumor cell proliferation, apoptosis, migration, and invasion by binding to target genes [113]. MiR-375 is also an important regulator of the EMT process, inhibiting the development of tumor cells by affecting the relevant target genes of EMT (Figure 2). Moreover, lncRNAs and circRNAs also affected the role of miR-375 in tumors via the ceRNA mechanism (Table 2). Therefore, miR-375 is being explored as a new type of cancer therapy [17]. With the continuous development of new technologies, various miRNA delivery vectors have been used in various animal experiments, with significant results [114]. However, there are still some challenges that need to be considered. For example, although nanocarriers have the advantages of low toxicity and high accumulation in tumors, the long-term effects are still unknown, and the specific regulatory mechanism of miR-375 *in vivo* is still unclear [110,115]. Although there are many problems that still await solutions, current studies indicate that miR-375 could be a suitable candidate for cancer treatment and also serve as potential tumor biomarkers to improve diagnostic efficiency.

## Abbreviations

AC: adenocarcinoma
AKT: protein kinase B
ASH1: achaete-scute homolog-1
BC: breast cancer
circRNA: Circular RNAs
ceRNA: competitive endogenous RNAs
CBX3: chromobox 3
COAD: colon adenocarcinoma
CRC: colorectal cancer
DOX: doxorubicin hydrochloride
EMT: epithelial–mesenchymal transition
ER: estrogen receptor
FGF: Fibroblast growth factor
HGF: hepatocyte growth factor
HIF: hypoxia-inducible factor-1
JAK2: Janus kinase 2
lncRNA: Long non-coding RNAs
miRNA: microRNA
MM: multiple myeloma
NPC: nasopharyngeal carcinoma
NSCLC: non-small cell lung cancer
OS: osteosarcoma
PC: prostate cancer
PDPK1: phosphoinositide-dependent protein kinase 1
PTC: papillary thyroid carcinoma
RASD1: Ras-related dexamethasone-induced 1
SCC: squamous cell carcinoma
SCLC: small cell lung cancer
SHOX2: stature homeobox 2
SNHG17: small nucleolar RNA host gene 17
SP1: Specificity protein 1
STAT3: signal transducer and activator of transcription 3
TEAD: transcriptional enhanced associate domain
TGF-β: transforming growth factor β
YAP1: Yes-associated protein 1
YWHAZ: tyrosine 3-monooxygenase/tryptophan 5-monooxygenase activation protein ζ
